# Insights Into the Involvement of Circular RNAs in Autoimmune Diseases

**DOI:** 10.3389/fimmu.2021.622316

**Published:** 2021-02-25

**Authors:** Xingyu Zhai, Yunfei Zhang, Shuyu Xin, Pengfei Cao, Jianhong Lu

**Affiliations:** ^1^Department of Hematology, Xiangya Hospital, Central South University, Changsha, China; ^2^National Healthcare Commission Key Laboratory of Carcinogenesis, Department of Microbiology, School of Basic Medical Science, Central South University, Changsha, China; ^3^China-Africa Research Center of Infectious Diseases, Central South University, Changsha, China; ^4^Center for Medical Experiments, The Third Xiangya Hospital, Central South University, Changsha, China

**Keywords:** circRNA, biomarker, function, autoimmune response, autoimmune diseases

## Abstract

Circular RNAs (circRNAs) are single-stranded, endogenous, non-coding RNA (ncRNA) molecules formed by the backsplicing of messenger RNA (mRNA) precursors and have covalently closed circular structures without 5′-end caps and 3′-end polyadenylation [poly(A)] tails. CircRNAs are characterized by abundant species, stable structures, conserved sequences, cell- or tissue-specific expression, and widespread and stable presence in many organisms. Therefore, circRNAs can be used as biomarkers for the prediction, diagnosis, and treatment of a variety of diseases. Autoimmune diseases (AIDs) are caused by defects in immune tolerance or abnormal immune regulation, which leads to damage to host organs. Due to the complexity of the pathophysiological processes of AIDs, clinical therapeutics have been suboptimal. The emergence of circRNAs sheds new light on the treatment of AIDs. In particular, circRNAs mainly participate in the occurrence and development of AIDs by sponging targets. This review systematically explains the formation, function, mechanism, and characteristics of circRNAs in the context of AIDs. With a deeper understanding of the pathophysiological functions of circRNAs in the pathogenesis of AIDs, circRNAs may become reasonable, accurate, and effective biomarkers for the diagnosis and treatment of AIDs in the future.

## Introduction

Immunity is a physiological protective mechanism that is inherent to the body. Through the immune system, the body recognizes antigenic substances, distinguishes “self” from “non-self” components, performs immune surveillance, and defends against the invasion of pathogens to eliminate antigenic substances from the body and maintain health (1–3). The function of immunity mainly includes three aspects, immune surveillance, defense, and stability, which may participate in antitumor, antibacterial, and antiviral activities (4, 5). Immunity is usually beneficial to the organism but, when defective, causes a pathological immune response. For example, low immune surveillance function may lead to the formation of tumors, but a stable high immune function may lead to autoimmune diseases (AIDs), such as systemic lupus erythematosus (SLE). Low defense function can cause immune deficiency syndrome, but the opposite condition can cause allergic reactions.

In general, a normal body can recognize its own tissue components without producing an immune response, which is called self-tolerance. The breakdown of self-tolerance produces a mass of autoreactive T lymphocytes or autoantibodies and causes an immune response against the body's own components, which is termed autoimmunity (6). Loss of immune tolerance or abnormal immune regulation leads to the destruction of self-tissues, which causes various AIDs (7).

Due to the complexity of the pathophysiological processes of AIDs, clinical therapeutics have been suboptimal. Thus, for decades, many researchers have been conducting research for biomarkers to be used in the diagnosis and treatment of AIDs. At present, some traditional protein biomarkers have been used in clinical diagnosis and treatment, but the results are not satisfactory. With the development of the chip technology and high-throughput sequencing, the mechanism and biological effects of circular RNAs (circRNAs) in AIDs have been gradually discovered. CircRNAs are single-stranded, endogenous, non-coding RNA (ncRNA) molecules formed by the backsplicing of messenger RNA (mRNA) precursors and have covalently closed circular structures without 5′-end caps and 3′-end polyadenylation [poly(A)] tails. Due to their conservation, stability, and specificity of structure, circRNAs have quickly become a hot spot (8) and are expected to become biomarkers for the prediction, diagnosis, and prognosis of diseases.

### CircRNAs—Past and Present

In 1976, Sanger first discovered a single-stranded, endogenous, ncRNA formed by covalent bonds in plant RNA viruses (9, 10). Subsequently, in 1979, Hsu and Cocaprados used electron microscopy to detect non–free-ended circRNAs in the cytoplasm of HeLa cells. At the time, it was wrongly assumed that circRNAs were simply the product of faulty splicing (11). CircRNAs were first identified in fungi by Matsumoto et al. (12). In the early 1990s, Nigro et al. found that non-classical splicing transcription occurred in human colon cancer gene, human EST 1 gene, and mouse Sry gene, but they believed that these covalently closed circRNAs were the products of incorrect connections although they still played a role (13–16). In 1996, it was found that circRNAs could be produced *in vitro* from nuclear extracts (17, 18). From the late 1990s to the early twentieth century, studies found that the rat cytochrome *P450 2C24* gene (19), the human dystrophin gene (20), and other genes could produce circRNAs. Although these studies proved the presence of circRNA molecules, researchers did not fully understand their potential impact. In 2010, several studies found thousands of circRNAs in multicellular animals that are specifically expressed in tissues and spatiotemporally expressed during development (21–23). In 2018, Toptan et al. also found circRNAs of viral genetic origin in Epstein–Barr virus (EBV) and Kaposi's sarcoma virus (24). In recent years, with the development of bioinformatic analysis technology, high-throughput sequencing technology, special computational pipeline development, and circRNA detection methods (25, 26), circRNAs have truly entered human vision and have become a hot spot in the scientific research field.

## The Formation of circRNAs

**Figure 1:**
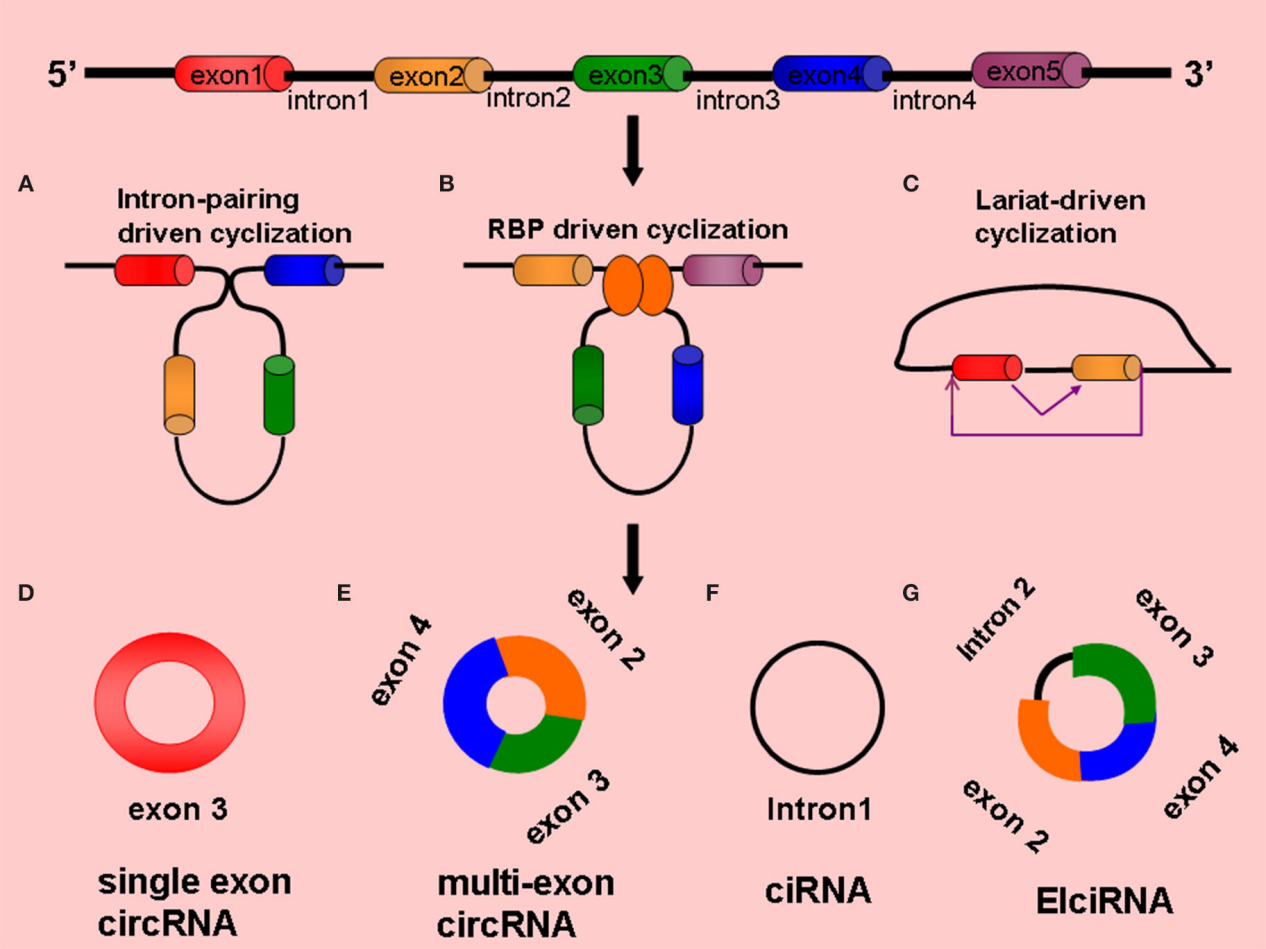
Formation and types circRNAs **(A)** Intron-pairing driven cyclization, **(B)** RBP-driven cyclization, **(C)** Lariat-driven cyclization, **(D)** Single exon circRNA: circRNA composed of a single exon, **(E)** Multi-exon circRNA: circRNA composed of multiple exons, **(F)** CiRNA: circRNA composed of introns, **(G)** ElciRNA: circRNA composed of introns and exons.

Circular RNAs are single-stranded, endogenous, ncRNA molecules formed by the backsplicing of mRNA precursors and have covalently closed circular structures without the 5′ end caps and 3' poly(A) tails (27). According to different sources, circRNAs can be divided into single-exon circRNAs, multiexon circRNAs, exon-intron circRNAs (EIciRNAs), and intron-only circular intronic RNAs (ciRNAs); the first two types are located in the cytoplasm, and the last two types are located in the nucleus (28) (Figure 1). The same gene can generate different circRNAs through alternative splicing, which is involved in the regulation of different biological processes. The available evidence suggests that circRNAs are not the product of mere accidents of splicing (22, 27). The complete mechanism of the formation of circRNAs has not yet been clarified.

### Intron-Pairing Driven Cyclization

The flanking introns of some exons contain reverse repeat sequences [e.g., Alu elements and reverse complementary paired sequences (RCMs)], and when these sequences are close together, they are paired with complementary bases to form circRNAs by variable splicing (29), with the circRNAs generally containing 2–3 exons. For example, the human *POLR2A* gene can generate a certain circRNA that contains two exons. The introns on the flanks of the two exons contain two complementary Alu elements, and different types of circRNAs can be formed by variable shearing, which cannot occur when the Alu elements are removed (22, 25). Studies have shown that the same characteristic element was found in the lateral intron region of *Caenorhabditis elegans* and human circRNAs, which are enriched with RCMs that complement each other at the splice site to form RNA double-stranded bodies, which are then formed into circRNAs by variable splicing (30, 31) (Figure 1).

### RNA-Binding Proteins Driven Cyclization

RNA-binding proteins (RBPs) can also regulate the biogenesis of circRNAs. When an RBP that binds to an exon's lateral and intron region undergoes dimerization, the upstream splicing acceptor site (SA) and downstream splice donor site (SD) bind to each other, and the exon undergoes reverse splicing and generates a circRNA (32, 33). For example, RNA-binding protein FUS (Fused in Sarcoma)-mediated reverse splicing of RNA forms circRNAs in mouse neurons (34). In the process of epithelial–mesenchymal transformation, quaking (QKI) proteins can bind to introns on the flanks of the exons, orienting them side by side to promote cyclization (33). In *Drosophila melanogaster*, intronic repeats, heterogeneous ribonucleoproteins, and SR proteins play roles in the formation of circRNAs (35). Musclebind (MBL) proteins can also bind to introns on the exon flanks for dimerization, which promotes the formation of circRNAs (36) (Figure 1).

### Lariat-Driven Cyclization

The lariat-driven cyclization mechanism is the earliest and most common method of cyclization (37); in this mechanism, the covalent binding of splicing donors and splicing acceptors by exon-skipping reading occurs to form cyclization (38). Jeck et al. proposed a lariat-driven cyclization model formed by exon circRNAs based on this mechanism, believing that pre-mRNA can generate a lariat structure containing exons through exon-skipping events and can then remove intron sequences by lariat structure splicing to produce circRNAs (22, 26) (Figure 1).

### Other Factors Affecting Cyclization

An increasing number of studies has shown that the formation of circRNAs is also affected by a variety of factors. For example, the spliceosome E complex assembled on exons of yeast EFM5 and HMRA1 was able to induce pre-mRNA backsplicing to form circRNAs when exons were long enough (39). The immune response factors NF90 and NF110 can stabilize the double-stranded RNA structure formed during transcription and can facilitate the formation of circRNAs by backsplicing (40). During the development of germ cells of male mouses, m6A modification can promote the formation of open reading frames (OFR)-carrying circRNAs (41). Numerous studies have found that endonuclease Cpsf73, SRSF3 splicing factor, pathogenic gene *RBM20*, and *Csy4* can regulate the occurrence of the formation of circRNAs (42–45).

## Degradation of circRNAs

**Figure 2:**
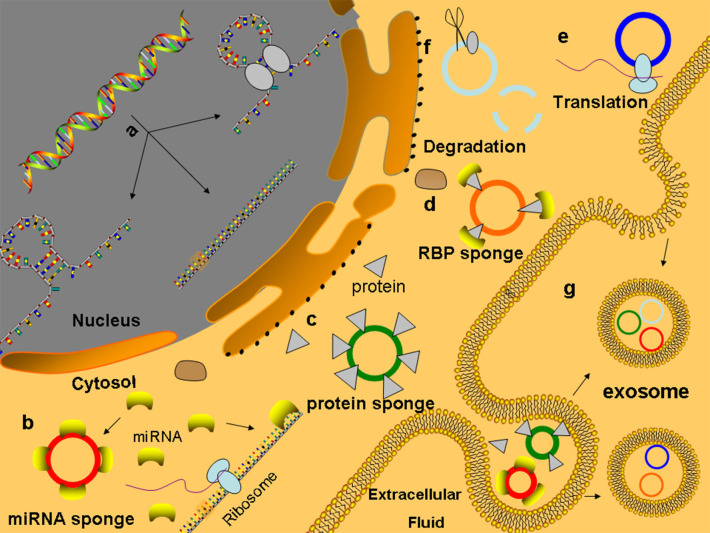
Functions of circRNAs **(a)** CircRNAs are formed and processed in the nucleus. **(b)** MiRNA sponge: circRNAs contain many MREs to competitive combination of microRNAs, which can competitively inhibit the binding of mRNA to microRNAs to regulate the expression of genes. **(c)** CircRNA can adsorb proteins like sponge adsorption of microRNAs and regulate the activity of protein. **(d)** CircRNAs combined with proteins act as the bracket of RBP, provide a platform for interactions between proteins and DNA, RNA, or proteins, and rapidly respond to extracellular stimuli **(e)** CircRNAs can be translated into protein-like linear RNA. **(f)** CircRNAs containing m6A can bind and degrade through the YTHDF2–HRSP12-mediated RNA endonuclease RNase P/MRP complex. **(g)** CircRNAs can enter the exosomes to protect themselves from being cleared and play a role through exosome transfer to target cells.

Currently, the relevant mechanisms of the degradation of the circRNAs are very limited. Due to the lack of a 3′ poly (A) tail and 5′ end cap, circRNAs are not easily degraded by RNA enzymes, so their degradation is different from the linear RNA degradation mechanism (42–45). At present, several related degradation mechanisms have been identified. (a) In m6A-mediated RNA degradation, circRNA molecules that contain m6A can bind to and be degraded by the YTHDF2-HRSP12-mediated RNA endonuclease RNase P/MRP complex (46). (b) There may be a degradation pathway for circRNAs similar to the structure-mediated RNA decay (SRD) mechanism for mRNA, which is degraded by the RBP UPF1 and its associated protein G3BP1 pathway (47). Studies have shown that the middomain in the protein sequence of GW182 plays a key role in the degradation of circRNAs (48) (Figure 2).

## Biological Functions of circRNAs

### CircRNAs Acting as miRNA Sponges

A large number of studies have shown that circRNAs are rich in miRNA response elements (MREs) that can sponge absorbed miRNAs, inhibit the binding of miRNAs to target genes, and thus regulate the expression of target genes. This mechanism is known as the regulation hypothesis of competing endogenous RNA (ceRNA) (49, 50). CircRNAs that contain multiple competitive binding sites and are not easily degraded, which have been found in many tissues, are more likely to perform the functions of ceRNA (8) (Figure 2). Human circRNA CDR1as (CIRS-7, hsa_circ_0001946), which is a representative circRNA, has ~74 miR-7 binding sites (63 are conserved). Although hsa_circ_0001946 is densely bound by miR-7, hsa_circ_0001946 is not silenced by miR-7. Both hsa_circ_0001946 and miR-7 are highly expressed in brain tissues. Hansen et al. proved that hsa_circ_0001946 reduces the knockdown efficiency of miR-7, while the transfection of miR-671, which reduces the CDR1as levels before introducing miR-7, recuses the sensitivity of the evaluated target genes to miR-7. In patients with SLE, hsa_circ_0012919-bound miR-125a-3p competitively mediated the gene expression of the target proteins RANTES and KLF13, causing acute and chronic inflammatory pathophysiological processes (51, 52). Analogously, in peripheral blood monocytes (PBMCs) of patients with multiple sclerosis (MS), hsa_circ_0001742 competitively binds to miR-634, which is transcribed from PRKCA gene introns, thus regulating the PRKCA expression (Table 2). In addition, the authors identified 16 putative miR-138 target sites in the testis-specific circular Sry RNA, suggesting that Sry RNA is an miR-138 sponge (78).

Studies have found that some circRNAs can bind to proteins, such as through sponge adsorption of miRNAs, and then regulate the activity of these proteins. For example, after the competitive combination of circSamd4 (mmu_circ_0000529) and the PUR protein, the myogenic transcriptional activity of the purine-rich binding proteins (PUR) protein was inhibited, and the transcription of myosin heavy chains was promoted to enhance the process of muscle generation (79). CircACC1 (hsa_circ_001391) can directly interact with the β and γ subunits of adenosine monophosphate activated protein kinase (AMPK) to promote AMPK stability and tumor growth (80). CircAmotl1 (hsa_circ_0004214) can combine with PDK1 and AKT1, thereby reducing cell apoptosis and promoting cardiac repair (81).

### The Involvement of circRNAs in the Regulation of the Gene Expression

Many studies have indicated that circRNAs play an important role in the regulation of the gene expression (Figure 2). The biogenesis of circRNAs negatively affects the efficiency of pre-mRNA alternative splicing and exerts regulatory effects on the gene expression (82). Recent studies show that there is a competition between backsplicing and linear splicing due to overlapping dependence on the spliceosomal machinery (83). CircMbl (hsa_circ_0030647) is cyclized from the second exon of the splicing factor MBL/MBNL1 in flies and humans, and its flanking introns contain conserved muscleblind binding sites, which are strongly and specifically bound by MBL. The modulation of the levels of MBL strongly affects the biosynthesis of circMbl, and downregulation of MBL in both cell culture and fly neural tissue leads to a strong and significant decrease in the production of circMbl. If the MBL protein level is in excess, its mRNA is downregulated by increasing the production of circMbl. Together, the above data suggest that circRNAs have a conserved function in gene regulation by competing with canonical splicing (36). In the biogenesis of circRNAs, the pre-mRNA backsplicing process is generally coupled to alternative splicing. It is still unclear how the splicing machinery selects either alternative splicing or backsplicing to generate a circular RNA. CircRNAs also suppress parental gene transcription by occupying RNA binding sites in target genes. The SEP3 exon 6 circRNA strongly binds to its cognate DNA locus and blocks the binding of its linear isoform to cognate DNA. The formation of RNA: DNA hybrids or R-loops results in the termination of SEP3 gene transcription (84). This phenomenon can directly regulate the levels of other RNAs through the ceRNA mechanism, such as the interaction of circPAIP2 (hsa_circ_0001538) with RNA polymerase II (Pol II) and U1 small ribonucleoprotein particles to enhance the transcription of the parental gene PAIP2 (85, 86). Unmodified circRNAs directly activate RIG-I in the presence of lysine-63-linked polyubiquitin chain to cause the filamentation of Mitochondrial Anti-Viral Signaling protein (MAVS) and the activation of the downstream transcription factor IRF3 to induce the production of interferon (87). For example, in breast cancer, circRNA-FECR1 (hsa_circ_0024836) can participate in the regulation of the methylation modification of CpG islands in the promoter region to activate the parental gene FLI1 and interact with TET1 and trans-DNMT1 to coordinate DNA methylation and demethylation to promote the development of tumors (88).

### Cooperative Actions of circRNAs Interacting With Proteins

Research suggests that circRNAs may be involved in the assembly of RNA complexes or proteins. It is also possible to combine multiple proteins to act as scaffolds of RBPs, forming a molecular library of proteins that provides a platform for interactions between proteins and DNA, RNA, or proteins, thus producing a rapid response to extracellular stimulation (Figure 2). CircMRPS35 (hsa_circ_0000384) recruits histone acetyltransferase KAT7 to the promoters of FOXO1, FOXO3a, and acetylated H4K5 and induces the expression of its downstream target genes p21 and E-cadherin, which promotes tumor immunity and inhibits the proliferation and invasion of gastric cancer cells (89). Upon virus invasion, the antiviral protein NF90/NF110 is transported from the nucleus into the cytoplasm where it binds to circRNAs to form a molecular reservoir of Nf90/Nf110 and leads to a global decrease in the level of circRNAs (90). Circ-DNMT1 (hsa_circ_0049224) interacts with p53 and AUF1 to promote the complex to enter the nucleus, and the AUF1 protein cannot inhibit DNMT1 mRNA after entering the nucleus, leading to the accumulation of the DNMT1 protein, which inhibits p53 transcription, and the promotion of the gene expression related to tumor proliferation (91).

### Functions of Translated circRNAs

In addition to the above functions, circRNAs may also be translated into proteins. Traditionally, the 5′-cap and 3′-poly(A) tails are essential elements that initiate and drive translation. Interestingly, researchers demonstrated that circRNA translation can promote cap-independent translation after m6A modification in the 5′ untranslated region (92, 93) and can also activate the translation mechanism by binding ribosome to circRNAs through internal ribosome entry site (IRES) (94, 95) (Figure 2). For example, circAkt3 (hsa_circ_0000199) can encode protein 174-aa, which interacts with activated PDK1 to inhibit the phosphorylation of Akt at Thr308, thereby regulating the PI3K/Akt signaling pathway (96, 97). circβ-catenin (hsa_circ_0004194) from the source of the oncogene catenin was able to encode the 370-amino acid β-catenin isoform. This amino acid inhibits the phosphorylation of GSK3 and leads to the degradation of β-catenin, which in turn leads to the continuous activation of the Wnt/β-catenin pathway and promotes the development of liver cancer (98).

## The Role of circRNAs in Immunity

**Figure 3:**
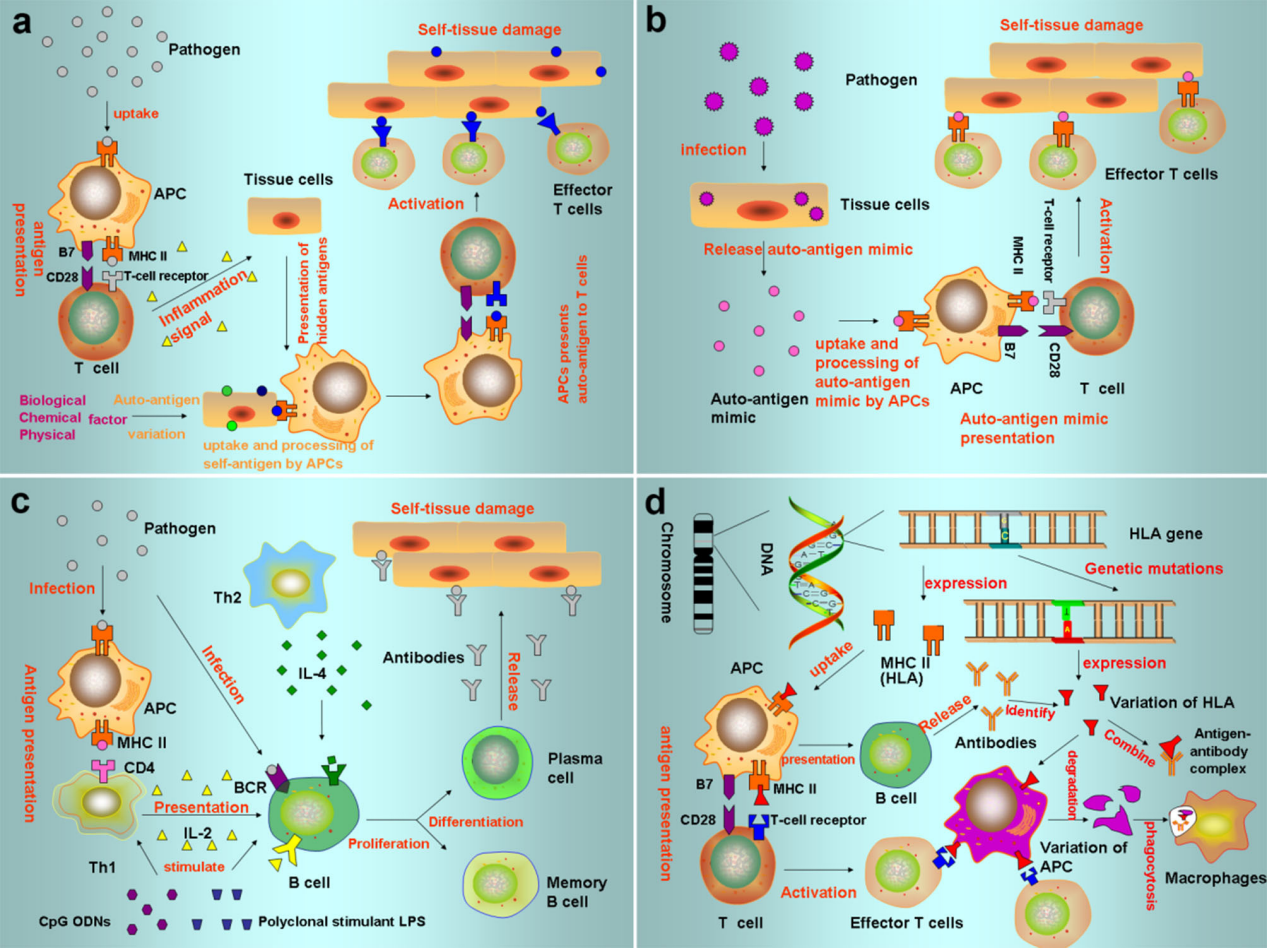
Pathogenesis of autoimmune diseases **(a)** Autoantigen variation: the tolerance genes in the body are mutated by biological, chemical, and physical factors, resulting in new antigen determination clusters or previously hidden antigen epitopes present autoreactive T cells, leading to autoimmune response. **(b)** Cross immunoreaction: the antigen carried by the pathogen is the same as the auto-tissue antigen, which can stimulate the body to produce common antibodies and bind to the same epitope of different antigens, causing autoimmune diseases. **(c)** Abnormal regulation of immune responses: after the invasion of polyclonal stimulants (Polyclonal stimulant LPS, CpG ODNs) and pathogens, Th cells that recognize the antigenic determinant of the body's own components are still in a tolerant state, while Th cells that recognize the foreign antigenic determinant are activated to helper B cells, produce an immune response, and cause an autoimmune reaction. **(d)** Genetic factors: HLA genes can express MHC. When HLA genes are mutated, the abnormal expression produces mutated HLA antigen, which causes autoimmune responses.

The immune system can recognize antigenic substances, distinguish “self” from “nonself” components, perform immune surveillance, and defend against the invasion of pathogens to maintain the physiological stability and balance of the internal environment of the organism (1–3). Therefore, the accurate regulation of the expression of genes of the immune system is critical to an organism's ability to generate powerful immunity to pathogens while limiting autoimmunity toward self-antigens (99) (Figure 3). CircRNAs have now been demonstrated to be active participants in this process, participating in pathogen-response pathways and in multiple stages of immunity.

Recent studies provide strong evidence that circRNAs are key regulators of immunity. Some exogenous circRNAs can activate immune gene expression and an antiviral program in organisms (100), while endogenous circRNAs can collectively inhibit double-stranded RNA (dsRNA)-activated protein kinase (PKR) and set the threshold for innate immunity upon virus infection (101). Pattern recognition receptors (PRRs) are critical to the mammalian innate immune system and recognize pathogen-associated molecular patterns (PAMPs) that are common among viruses and bacteria. RIG-I and PKR are PRRs that discriminate endogenous and exogenous circRNAs. Intriguingly, when circRNAs were synthesized in cells instead of being synthesized *in vitro* and transfected into cells, the immune stimulatory activity was dependent on the splicing mechanism. CircRNAs generated with endogenous introns and spliced by cellular spliceosomes did not stimulate RIG-I (100). In contrast, circRNAs made with self-splicing introns (phage td intron) stimulated RIG-I. This is because m6A modification abrogates adjuvant activity and immune gene activation. However, unmodified circRNA adjuvant induces antigen-specific T-cell and B-cell responses, and it directly activates RIG-I in the presence of lysine-63-linked polyubiquitin chain to cause filamentation of the adaptor protein MAVS and activation of the downstream transcription factor IRF3. Furthermore, PKR recognizes dsRNA in the cytoplasm and then inhibits protein synthesis. Previous work has shown that endogenous circRNAs tend to form 16 to 26 bp RNA duplexes. These dsRNAs (102, 103) and the adenovirus small non-coding VAI RNA (104) can act as inhibitors of dsRNA-activated PKR related to innate immunity. Upon viral infection or poly (I:C) stimulation, circRNAs are globally degraded, leading to PKR activation during early innate immune responses. In addition, circRNAs are closely associated with the immune factors NF90/NF110 in innate immunity. NF90 and NF110, which are both splice variants from the common gene ILF3, are dsRNA-binding proteins that are formed by inverted repeat elements in the introns flanking the two splice sites. These splice variants stabilize the RNA hairpin structure that promotes backsplicing. Upon virus invasion, NF90/NF110 is transported from the nucleus into the cytoplasm, which leads to a global decrease in the level of circRNAs. Moreover, NF90/NF110 can be released from the complex and can bind to a viral mRNA to inhibit viral replication (90, 105). Hsa_circ_0020397 can promote the expression of programmed death-ligand 1 (PD-L1) and telomerase reverse transcriptase by binding to miR-138, thereby regulating the apoptosis, viability, and invasion of colorectal cancer cells. PD-L1 is closely related to tumor escape from immune control, so hsa_circ_0020397 may promote tumor development by regulating tumor immunity (106). During the process of immunosenescence, there is a significant feature, namely, that the proportion of CD8 T lymphocytes lacking CD28 expression would be increased. Studies indicate that hsa_circ_100783 in aging human CD8+ T cells might function as a new biomarker for CD28-related CD8+ T-cell aging (107). Collectively, these results provide strong evidence that circRNAs are key regulators of immunity.

## The Involvement of circRNAs in AIDs

Emerging evidence suggests that circRNAs play important roles in complex human pathology. It is worth noting that circRNAs are important gene regulators in the immune system and are closely related to the occurrence and development of AIDs. Here, we focus on the role of circRNAs in immune cells and immune regulation and emphasize their potential as biomarkers and biological functions in autoimmunity.

### The Role of circRNAs in Systemic Lupus Erythematosus

Systemic lupus erythematosus is a kind of disease in young women (108) that can cause the production of predominantly antinuclear antibodies, among a variety of autoantibodies, and is an autoimmune inflammatory connective tissue disease that involves multiple organs (109). SLE is characterized by complement failure, autoantibodies, immune complex deposition, and frequent inflammation recurrence (110–112) (Table 1).

**Table 1:** Types and characteristics of autoimmune diseases.

**Autoimmune diseases**	**Vulnerable population**	**Clinical symptoms**	**Pathogenesis**	**References**
Systemic lupus erythematosus	20–40 years old female	Fatigue, fever, butterfly erythema, arthritis, pericarditis, pleurisy, pulmonaryembolism, nephritis, aseptic meningitis, lupus like sclerosis, pancreatitis, hyperthyroidism or hypothyroidism	Under the interaction of environmental and genetic factors inhibiting function of T cells is reduced, the decrease in the number of T cells, B cells excessive proliferation of antinuclear antibodies, and result in granulocyte cell membrane damage or degradation, swelling of the nucleus, the formation of lupus corpuscle, LE corpuscle induce macrophages and neutrophils chemotaxis, combined with the formation of the antibody specificity immune complex, deposited on the skin, blood vessels, joints. In the presence of the complement, lupus cells form, leading to inflammation and tissue necrosis. Or directly through autoantibodies and tissue cell antigen interaction, causing cell damage.	(108–112)
Rheumatoid arthritis	40–60 years old female	Joint capsule injury, vasculitis, pleurisy, subcutaneous nodules, rheumatoid nodules, serosal inflammation, pneumonia, lymphadenopathy, splenomegaly, leukopenia, lymphatic follicular formation	Virus (EBV), bacteria and other infectious factors enter the body, its oligosaccharides, glycopeptide fragments and other components are absorbed by synovium cells to form new proteoglycan, change the structure of IgG, and promote the body to produce specific antibodies-rheumatoid factor. IgM, IgG, IgA and IgE etc. can combine with RF to form immune complexes, which can be deposited in joints or local tissues, activate the complement and produce C3a and C5a, prompting neutrophils and monocytes to englobe RF-IgG and other immune complexes, synthesize and release lysosomal enzymes and IL-1 and other mediators, thereby causing the occurrence of RA.	(113–116)
Multiple sclerosis	20–40 years old female	Eye muscle paralysis neuritis, retrobulbus optic neuritis, limb paralysis, mental symptoms, limb tremor, nystagmus, and ataxia	Autoreactive T cells as being involved, particularly through their secretion of cytokines and activation of the inflammatory cascade.	(117–122)
Primary biliary cirrhosis	Middle-aged females	Pruritus, fatigue, jaundice, dyslipidemia	Mostly unknown; maybe related to autoimmunity.	(123–125)
Progressive systemic sclerosis	Common in women, with incidence of about 4 times than that of men, rare in children	Raynaud's phenomenon, skin involvement, musculoskeletal injury, and various degrees of visceral fibrosis	Under the influence of genetic, environmental factors and immune abnormalities, fibroblasts may synthesize and secrete more collagen, leading to fibrosis of skin and internal organs. Or RF, anti-smooth muscle antibody forms antigen antibody immune complex and deposits in the blood vessels and other organs, causing vascular endothelial cell injury, vascular wall fibrosis, and tissue fibrosis	(126, 127)
Rheumatic heart disease	Young people	Palpitation, shortness of breath, cough, fatigue, lower limb edema Atrial fibrillation	RHD refers to valvular heart disease caused by abnormal autoimmune response after infection with group A type b hemolytic streptococcus.	(128–130)
Psoriasis	Young adults	Erythema, scales, pruritus, joint pain	Unknown	(131–133)
Lupus nephritis	Young adults	Hematuria, proteinura, edema, chronic renal failure	The pathogenesis of LN maybe associated with circulating immune complex deposition in the kidney;	(134–136)

**Figure 4:**
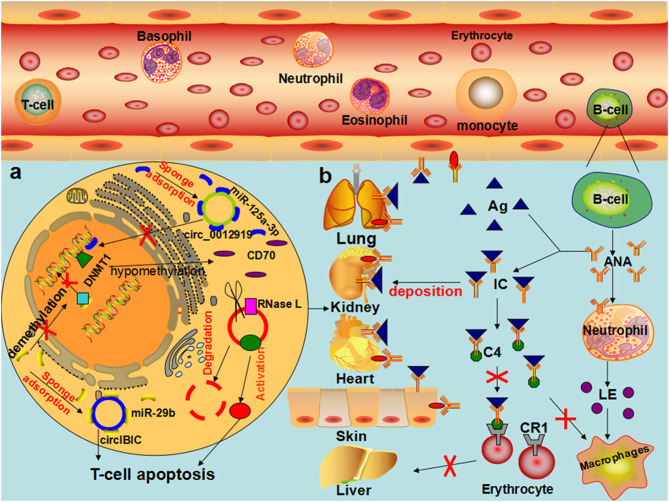
The role of circRNAs in the pathophysiological process of SLE **(a)** In T cells of patients with SLE, hsa_circ_0012919 competitively binds to mir-125a-3P through ceRNA mechanism, regulates the expression of DNMT1/RANTES gene, reduces the level of CD70, and causes the pathophysiological process of acute and chronic inflammation, leading to the occurrence and development of SLE. CircIBTK competitively binds to mir-29b, reverses mir-29b induced DNA demethylation, activates the AKT signaling pathway, and promotes T cell apoptosis. CircRNA is degraded by RNase L, and PKR enhances phosphorylation when the organism is infected by an associated virus or stimulated by Poly (I:C), leading to the development of SLE. **(b)** In patients with SLE, the decrease in the number of T cells and B cells with excessive proliferation of antinuclear antibodies result in granulocyte cell membrane damage or degradation, swelling of the nucleus, the formation of lupus corpuscle, the induction of macrophages and neutrophils chemotaxis by LE corpuscle, combined with the formation of the antibody specificity immune complex, deposited on the skin, blood vessels, and joints. In the presence of the complement, lupus cells form, leading to inflammation and tissue necrosis, directly through autoantibodies and tissue cell antigen interaction, causing cell damage.

Li et al. provided the first proof of the comprehensive expression profile of circRNAs in the plasma of patients with SLE to provide a basis for the use of circRNAs as biomarkers for the prediction and diagnosis of SLE (137). Subsequent studies showed that hsa_circ_407176, hsa_circ_001308, hsa_circ_0049224, hsa_circ_0044235, and hsa_circ_0068367 are clearly upregulated in the PBMCs of patients with SLE (138–140). Coincidentally, Luo et al. also found 11 significantly upregulated circRNAs in the PBMCs of patients with SLE (53). Interestingly, Li et al. demonstrated that hsa_circ_0045272 was significantly downregulated, which would result in certain miRNAs being free and the subsequent downregulation of NM_003466 (PAX8) and NM_015177 (DTX4) mRNAs, thereby negatively regulating the production and apoptosis of interleukin-2 (IL-2) in the T cells of patients with SLE (55). Our current knowledge is far too rudimentary to propose a mechanism of the occurrence and development of SLE mediated by circRNAs, but some researchers have performed preliminary explorations. Wang et al. demonstrated that downregulated circIBTK (hsa_circ_0077179) may reverse the DNA demethylation induced by miR-29b through competitive binding with miR-29b and activate the AKT signaling pathway in SLE (56). Research has shown that the PI3K/AKT pathway regulates macrophage migration and proliferation and coordinates the responses to different inflammatory signals in macrophages (141). AKT can also coordinate IL-2 signaling to maintain the expression of cytolytic effector molecules and cytokine and chemokine receptors in cytotoxic T cells (142). Zhang et al. discovered that hsa_circ_0012919 was downregulated by competitively binding to miR-125a-3p in patients with SLE, reversing the DNA hypomethylation of CD11a and CD70 in CD4+ T cells, increasing the expression of DNMT1, and reducing the expression of CD70 and CD11a. Subsequently, these authors found that hsa_circ_0012919 competitively binding to miR-125a-3p mediated the gene expression of the target proteins RANTES and KLF13, causing acute and chronic inflammatory pathophysiological processes (52, 57). Guo et al. confirmed that hsa_circ_0000479 influences the occurrence and development of SLE by regulating the Wnt signaling pathway and metabolic pathway (54). Bioinformatic analysis revealed that the parent gene of circPTPN22 (hsa_circ_0110529) is the regulatory factor of T cell activation. The downregulated circPTPN22 was correlated with mRNAs and miRNAs related to immune regulation and has a good diagnostic value for SLE (58). In addition, endogenous circRNAs were able to form inhibitors of dsRNA-activated protein kinase (PKR that participate in the antiviral innate immune response. When the body is infected with a relevant virus or stimulated with poly (I:C), circRNAs are degraded by RNaseL, and phosphorylation is enhanced by PKR, leading to the development of SLE (102, 103). Other evidence showed that hsa_circ_0000479 and hsa_circ_0082689 are correlated with the C3 levels and anti-dsDNA levels, respectively (53) (Table 2) (Figure 4). In the context of limited methods for the diagnosis and treatment of SLE, the circRNAs mentioned above may become potential biomarkers, and the specific mechanism associated needs to be further explored.

**Table 2:** Summary of the role of circRNAs in autoimmune diseases.

**CircRNA**	**Disease**	**Tissue**	**Expression pattern**	**Sponge target**	**Effect**	**References**
hsa_circ_0082689	SLE	PBMCs	**↓**		It is related to anti-dsdna level and treatment	(53)
hsa_circ_0000479	SLE	PBMCs	**↑**		The regulation of Wnt signaling pathway and metabolic pathway further affects the development of SLE	(53, 54)
hsa_circ_0045272	SLE	T cell	**↓**		Negatively regulates the production of interleukin 2 (IL-2) and promotes apoptosis of SLE T cells	(55)
hsa_circ_0077179	SLE	PBMCs	**↓**	miR-29b	Reverse the DNA demethylation induced by miR-29b, and activate the AKT signaling pathway, thus causing the occurrence and development of SLE	(56)
hsa_circ_0012919	SLE	CD4+Tcell		miR-125a-3p	Reverse DNA hypomethylation of CD11a and CD70 in CD4+ T cells. It regulates the gene expression of target proteins RANTES DNMT1 and KLF13, causing acute and chronic inflammatory pathophysiological processes that lead to the development of SLE	(52, 57)
hsa_circ_0110529	SLE	PBMCs	**↓**		The potential of SLE to diagnose biomolecular markers	(58)
hsa_circ_0035197, hsa_circ_0000175, hsa_circ_104194, hsa_circ_0001200, hsa_circ_0000396, hsa_circ_0130438	RA	PBMCs	**↑**		Potential biomarkers for RA diagnosis	(59–63)
hsa-circ-0001045	RA	Synovium	**↑**	miR-30a	It promotes the overexpression of beclin-1 and LC3 in RA, thereby reducing cell apoptosis and promoting the occurrence and development of RA	(64)
hsa_circ_0001859	RA	SW982 cell		miR-204/211	Inhibit the expression of ATF2 in RA, thereby reducing the chronic inflammation of synovial tissue	(65)
hsa_circRNA_100833	RA	Plasma	**↑**	miR-498	It acts as an inhibitor of miR-498/mTOR cross-talk by directly targeting miR-498, regulating ECM catabolism, inflammation, and apoptosis and affecting the pathophysiological process of RA.	(66)
hsa_circ_0001946	RA	Plasma	**↑**	miR-671 miR-7	The downregulation of miR-671 leads to the up-regulation of cirs-7,thereby reducing the inhibition of miR-7 on mTOR through the PI3K/AKT/mTOR signaling pathway, thus affecting the pathophysiological process of RA	(51, 66)
hsa_circ_0106803	MS	PBMCs		miR-149	Hsa_circ_0106803 can modulate the progression of MS by regulating the expression of ASIC1a mRNA through miR-149	(67–69)
hsa_circ_0005402	MS	PBMCs	↓	miR-1248 and miR-766	Through competitive binding of miR-1248 & miR-766, ANXA2 expression was regulated and thus involved in the occurrence and development of MS	(5, 70)
has_circ_402458	PBC	Plasma	**↑**	miR-522 and miR-943	It is a sponge of mir-522 and mir-943 that regulates inflammation-related signaling pathways, promotes the development of PBC	(71–73)
hsa_circRNA_001264, hsa_circRNA_104121, hsa_circRNA_045355	PSS				Potential biomarkers for PSS diagnosis	(74, 75)
circRNA chr2:206992521|206994966	Psoriasis	MSCs Plasma	**↓**	miR-7157-5p	It may play a role in the pathophysiological process of psoriasis by affecting the secretion of cytokines and the activity of local diseased T lymphocytes	(76)
circRNA_002453	LN	Plasma	**↑**		It is related to the severity of kidney damage; Potential biomarkers for LN diagnosis	(77)

***↓**, Downregulated; **↑**, Upregulated*.

### The Role of circRNAs in Rheumatoid Arthritis

Rheumatoid arthritis (RA) is a systemic AID that frequently occurs in young women and is chronic and recurring (113, 114). The immunological characteristics include synovial fluid and blood containing the rheumatoid factor (RF), inflammatory synovial fluid containing tumor necrosis factor (TNF-α), and other pro-inflammatory cytokines, and a large number of lymphocytes and macrophages in inflammatory synovial fluid (115, 116) (Table 1). Research has thoroughly documented that the inflammatory response and immunological disorders critically contribute to RA. However, the etiology and precise pathogenesis of RA remain to be fully elucidated.

**Figure 5:**
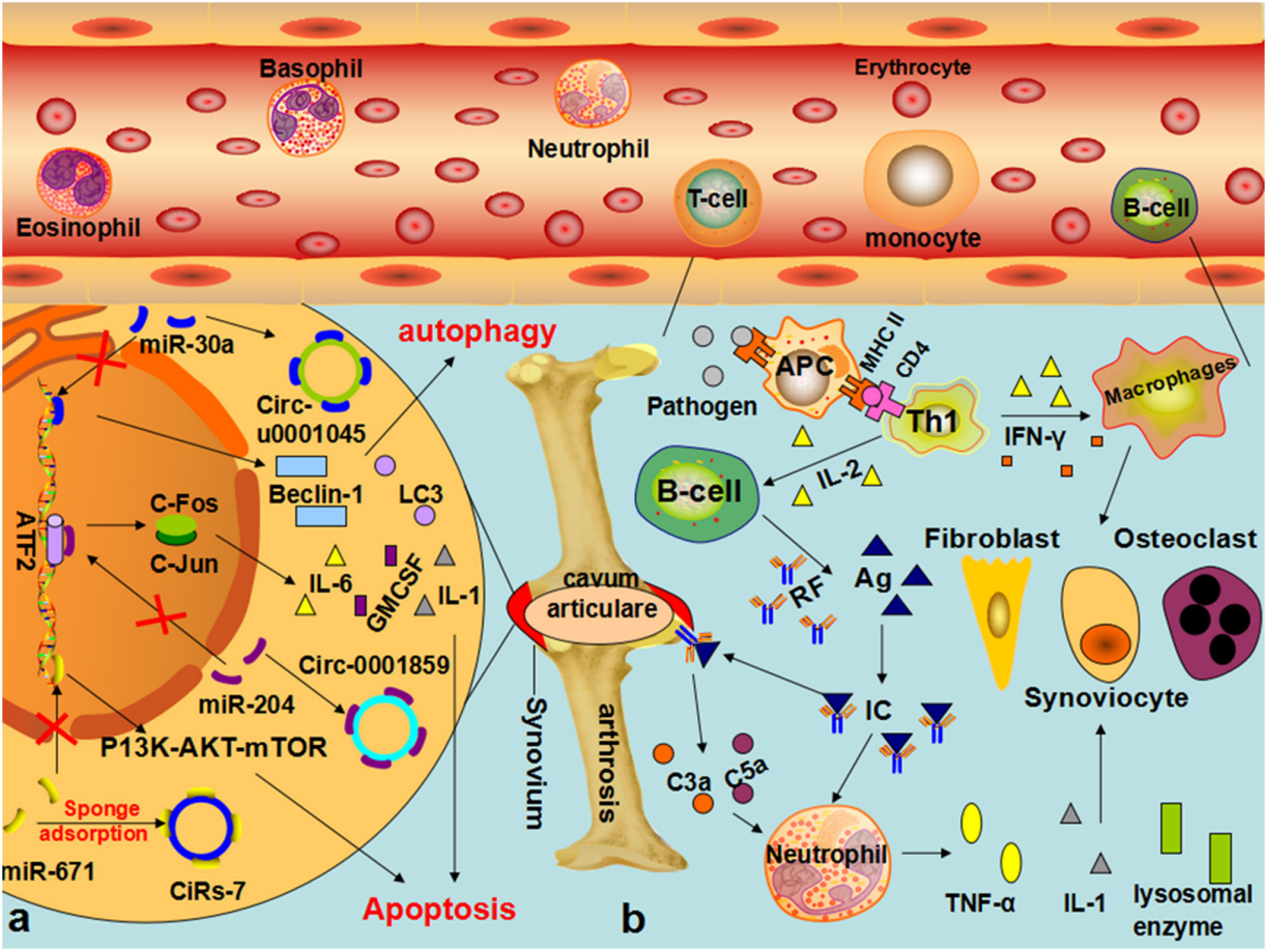
The role of circRNA in the pathophysiological process of RA **(a)** In synovial cells of patients with RA, hsa-circ-U0001045 competitively binds miR-30a, resulting in the overexpression of beclin-1 and LC3, promoting autophagy and the development and progression of RA. The downregulation of mir-671 resulted in the upregulation of CIRS-7, thereby reducing the inhibition of mir-7 on mTOR and affecting the pathophysiological process of RA through the PI3K/AKT/mTOR signaling pathway. **(b)** In patients with RA, EBV, bacteria, and other infectious factors enter the body, and its oligosaccharides, lipopeptide fragments, and other components are absorbed by synovium cells to form new proteoglycan, change the structure of IgG, and promote the body to produce a specific antibodies-rheumatoid factor. IgM, IgG, IgA, and IgE can combine with the RF to form immune complexes, which can be deposited in joints or local tissues, activate the complement, and produce C3a and C5a, prompting neutrophils and monocytes to englobe RF-IgG and other immune complexes, synthesize, and release lysosomal enzymes and IL-1 and other mediators, thereby causing the occurrence of RA.

Substantial research has discovered that circRNAs, such as hsa_circ_0035197, hsa_circ_0000175, hsa_circRNA_104194, hsa_circ_0001200, hsa_circ_0000396, and hsa_circ_0130438, are clearly dysregulated in RA (59–63). It is worth noting that hsa_circ_0002715 was correlated with the joint pain score, the joint swelling score, RF, and anti-imine protein antibodies (60). Hsa_circ_0000175 is correlated with the anti-citrate protein antibody and the count and percentage of white blood cells, lymphocytes, and neutrophils. Hsa_circ_0008410 is related to a joint range of motion, platelet count, and platelet deposition, indicating the range of motion and severity of RA. Li argued that hsa_circ_0044235 has diagnostic significance for RA and can also significantly distinguish patients with RA from patients with SLE (140). Our present knowledge is far too rudimentary to draft the mechanism of the occurrence and development of RA mediated by circRNAs, but some researchers have performed preliminary explorations. Xu argued for the first time that the increase in hsa-circ-0001045 in the synovial tissues of patients with RA significantly inhibits miR-30a, resulting in the overexpression of Beclin-1 and LC3 in RA and thereby reducing apoptosis and promoting the occurrence and development of RA (64). Li further demonstrated that hsa_circ_0001859 can inhibit the expression of ATF2 in SW982 cells after being silenced by competitive binding with miR-204/211, thereby reducing chronic inflammation in synovial tissue (65). In a recent study, hsa_circ_0088036 was found to promote the proliferation and migration of fibroblast-like synoviocytes by sponging miR-140-3p and upregulating SIRT 1 expression (143). More recent studies by Li et al. found that circFADS2 (hsa_circRNA_100833) acts as an inhibitor of miR-498/mTOR cross-talk by directly targeting miR-498, regulating extracellular matrix (ECM) catabolism, inflammation, and apoptosis, and affecting the pathophysiological process of RA. Coincidentally, with the downregulation of miR-671 in patients, miR-7 is correspondingly upregulated, which reduces the inhibition of mTOR by miR-7, and the activation of the PI3K/AKT/mTOR signaling pathway regulates the occurrence and development of RA (51, 66). Previous studies of the involvement of the circRNA–miRNA–mRNA ceRNA network in the pathophysiological processes of RA emphasized that it may become a potential biomarker for the diagnosis and treatment of RA (144) (Figure 5).

### The Role of circRNAs in Multiple Sclerosis

Multiple sclerosis is an autoimmune chronic inflammatory central nervous system (CNS) demyelination disease that frequently occurs in young women and is multifocal, transient, and prone to relapse (78, 121, 122) (Table 1). Studies about the pathophysiological mechanisms of MS, while not fully elucidated, have implicated autoreactive T cells as being involved, particularly through their secretion of cytokines and activation of the inflammatory cascade (117). Moreover, the inflammatory demyelination process triggers the microglial activation and chronic oxidative injury, leading to neurodegeneration and, ultimately, axonal and neuronal death (118). To date, the diagnosis of MS mainly depends on the clinical evidence of demyelinating lesions in the CNS. Cerebrospinal fluid (CSF) is the most direct source of biomarkers; however, multiple punctures are not recommended because of the invasiveness and potential adverse effects of the procedure, so CSF cannot be continuously sampled to monitor biomarker levels (117, 118). Additionally, the early symptoms of MS may be non-specific and suggestive of many other disorders of the CNS (119). Both the disease course and clinical phenotype of MS exhibit individual differences, and spatiotemporal variations occur even within the same individual. Although MRI can localize lesions to help diagnose them, it is expensive and difficult to perform. Because of the unique stability of circRNAs in body fluids, they have been proposed as promising sources of biomarkers in a wide variety of sample types, such as blood, saliva, urine, and CSF (120).

Paraboschi argued for the first time that the protein kinase C gene is associated with recurrent MS, but the underlying pathogenesis is still unknown (145). He et al. further demonstrated that the circRNAs (hsa_circ_0106803) formed by the fourth and fifth exons of Gasdermin B through reverse variable splicing were significantly dysregulated in the PBMCs of patients with Relapsing Remitting Multiple Sclerosis (RRMS) (69). Through bioinformatic analysis, it was predicted that hsa_circ_0106803 contains more than one target site, and among its binding partners, miR-149 and miR-1275 are differentially expressed in the blood of patients with MS. Previous studies have shown that miR-149 binds to ASIC1a and reduces its levels (67). ASIC1a encodes a subunit of the acid-sensing ion channel (68), which is overexpressed in lesions in acute MS and could be implicated in the neuronal pathogenesis of this disease. In light of this evidence, a ceRNA network has been proposed in which hsa_circ_0106803 can modulate the progression of MS by regulating the expression of ASIC1a mRNA through miR-149 (69). Subsequently, through the genome-wide association study of MS, it was also shown that hsa_circ_0043813 of STAT3 gene expression can be regulated by three disease-related SNP genotypes and can participate in the occurrence and development of MS (146). Moreover, another study of the PBMCs of patients with MS found that the expression of hsa_circ_0005402 and hsa_circ_0035560 encoded by the ANXA2 gene is downregulated (70). Through bioinformatic analysis, it was predicted that hsa_circ_0005402 contains a single binding site for 17 miRNAs and two binding sites for miR-1248 and miR-766 (5). Then, researchers performed a whole-transcriptome study with PBMCs from patients with LS-OCMBs and identified hsa_circ_0000478 (VWA8) and hsa_circ_0116639 (EP300), which are downregulated in patients with positive LS-OCMB status (120). Correale et al. used RNA-seq data to identify multiple differentially expressed circRNAs in sh-sy5y and Jurkat cells of MS-related tissues, including hsa_circ_0001329, hsa_circ_0006884, and hsa_circ_0006884 (78). In a recent study, Iparraguirre et al. used leukocytes from patients with MS to study the circular transcriptome. Among the differentially expressed circRNAs, 96.1% are upregulated in patients compared with controls. This suggests that the whole circRNA signature can have the potential to diagnose 85.2% of cases with MS (147). In the context of limited methods for the diagnosis and treatment of MS, the circRNAs mentioned above may become potential biomarkers. The process of determining whether they can be used in clinical diagnosis still has a long way to go.

### CircRNAs and Other AIDs

Primary biliary cirrhosis (PBC), which is also known as primary biliary cholangitis, is a chronic, organ-specific autoimmune liver disease with cholestasis (123, 125). The clinical manifestations are pruritus, fatigue, jaundice, and dyslipidemia (124). PBC is characterized by the presence of anti-mitochondrial antibodies in the circulating blood and the non-suppurative inflammatory destruction of the small intrahepatic bile duct, which leads to extensive destruction of the hepatic duct and biliary cirrhosis (124) (Table 1). The pathogenesis of PBC is unknown and may be related to autoimmunity. PBC often occurs in middle-aged women, with insidious onset, slow development, and mild early symptoms, making it difficult to diagnose (148). Therefore, it is urgent to identify biomarkers for the early diagnosis of PBC (149). Zheng et al. demonstrated 22 abnormally expressed circRNAs in the plasma of patients with PBC by circRNA microarray analysis. Subsequently, it was confirmed that hsa_circ_402458 could competitively bind to miR-522 and miR-943 (72). Previous studies have emphasized that miR-522 plays an important role in chronic inflammatory diseases (73), and miR-943 is involved in the occurrence and development of PBC by regulating TGF-livelihood signaling (71). Therefore, hsa_circ_402458 binds miR-522, and miR-943 competitively mediates inflammation-related signaling pathways, causing chronic inflammatory pathophysiological processes and becoming a potential biomarker for the early diagnosis of PBC (Table 2).

Scleroderma, which is also called progressive systemic sclerosis (PSS), is an inflammatory skin and internal organ fibrosis that is characteristic of chronic inflammatory connective tissue disease (126) (Table 1). Although significant progress has been made in the treatment of some AIDs, biomarkers for the prognosis of the therapy of PSS still need to be extensively studied (127). Su et al. identified 234 differentially expressed circRNAs in PSS. Subsequent studies showed that hsa_circ_001264, hsa_circ_104121, and hsa_circ_045355 are related to the pathophysiology of PSS and provide support for prognosis and treatment (74, 75) (Table 2).

Rheumatic heart disease (RHD) refers to a valvular heart disease caused by an abnormal autoimmune response after infection with group A type b hemolytic streptococcus. RHD is a complication of severe and recurrent rheumatic fever and often occurs in young people (128). The clinical symptoms are not obvious at the early stage, and symptoms of cardiac decompensation, such as palpitation, shortness of breath, cough, fatigue, and lower limb edema (129), appear later. Atrial fibrillation (AF) is common in patients with RHD. AF can be divided into permanent AF, persistent AF, and paroxysmal AF by duration. The main symptoms include palpitations, dizziness, chest discomfort, and shortness of breath (130). Through high-throughput sequencing, Hu et al. found 51 circRNAs with upregulated expression and 57 circRNAs with downregulated expression in patients with RHD persistent AF, verified the abnormal expression of circRNAs by qPCR, and conducted enrichment analysis of Gene Ontology (GO) and Kyoto Encyclopedia of Genes and Genomes (KEGG) pathways to construct the relevant circRNA–miRNA expression network. It was found that circRNA19591, circRNA19596, and circRNA16175 may competitively bind to miR-29b-1-5p and miR-29b-2-5p. This process is related to the pathogenesis of hypertrophic cardiomyopathy and dilated cardiomyopathy. CircRNAs may play a role in RHD-related AF (150, 151) (Table 2).

Psoriasis is an immune-mediated, heritable, chronic inflammatory skin disease (132). The disease mainly occurs in the skin and joints of young adults and is prone to relapse (133). Clinical manifestations are erythema, scales, pruritus, and joint pain (131) (Table 1). Therefore, the pathogenesis and diagnostic biomarkers of psoriasis urgently need to be studied. Qiao et al. demonstrated that many circRNAs are abnormally expressed in the pathological skin tissues of patients with psoriasis, and among these circRNAs, hsa_circ_0061012 may specifically bind to hsa-miR-7157-5p, hsa-miR-4769-3p, and hsa-miR-6817-5p, leading to psoriasis (152). Coincidentally, Moldovan et al. also found significantly downregulated expression of circRNAs in damaged skin tissues of patients with psoriasis (153). Subsequent studies showed that these significantly downregulated circRNAs were involved in the immune regulation by affecting the JAK-STAT pathway (154). Interestingly, research has demonstrated that circRNAchr2: 206992521|206994966 may play a role in the pathophysiological process of psoriasis by affecting the secretion of cytokines and the activity of locally diseased T lymphocytes (76) (Table 2). All these studies indicate that circRNAs have the potential to be biomarkers for the diagnosis and prognosis of psoriasis.

Lupus nephritis (LN) is a complication of SLE (136) and an immunological disease involving severe kidney damage (134). The pathogenesis of LN may be associated with circulating immune complex deposition in the kidney, local complement activation, and abnormal T cell-mediated immune response direct roles of autoantibodies, and other factors (135). The main clinical manifestations are hematuria, proteinuria, edema, and chronic renal failure (134) (Table 1). Early diagnosis of LN is critical because early detection and treatment can improve the prognosis of patients with LN (136); thus, the discovery of biomarkers for LN is urgently needed. Ouyang et al. detected the upregulated expression of circRNA_002453 in the plasma of patients with LN using the circRNA chip technology and found that it was related to the severity of renal injury and may become a potential biomarker for the diagnosis of LN (77) (Table 2).

## Conclusion and Future Perspectives

To date, the biological functions of most circRNAs remain poorly understood. However, a growing body of evidence suggests that circRNAs play important roles in the pathological physiology of numerous diseases by sponge adsorption, peptide coding, gene expression regulation, and epigenetic modification. These functions do not represent the global functions of all circRNAs but the functions of certain circRNAs because these functions are sequence-specific. However, in AIDs, a global change in the expression of circRNAs suggests a function that could also be global. Could a global change occur because many endogenous circRNAs tend to form imperfect RNA duplexes, which, *via* their interaction with the dsRNA-activated PKR, regulate the innate immune response (102, 103). To date, circRNAs mainly function through the circRNA–miRNA–mRNA ceRNA network to regulate gene expression and are involved in the occurrence of AIDs. Further research is needed to reveal whether circRNAs are involved in the pathophysiology of AIDs through other functions. However, circRNAs have the potential to serve as new clinical diagnostic biomarkers and therapeutic targets, providing the possibility for personalized precision medicine.

In recent years, circRNAs have attracted extensive attention in the prediction, diagnosis, and treatment of diseases, but there are still many problems to be solved. There is no standard nomenclature of circRNAs, although the study of circRNAs has exploded in the past few years, which is not convenient for academic exchanges (155). Many individual circRNA functions remain unknown due to the lack of adequate methods for distinguishing circRNAs from cognate mRNAs with overlapping exons (156). RNA sequencing (RNA-seq) data are used to detect genome-wide circRNA expression by numerous algorithms, but there is no clear standard way to evaluate the accuracy of these algorithms and little overlap in their predictions (157). Therefore, there is an urgent need for these problems to be studied.

It should be noted that there are limitations in the current studies on the role of circRNAs in AIDs. First, the number and sources of patient samples are limited, which affect the universality of circRNAs as biomarkers. Second, the level of circRNAs in patients has been determined, but the molecular mechanisms, such as protein translation and gene expression regulation, are still unclear and are thus regarded as a byproduct of splicing. Third, the prediction of circRNA targets, high-throughput sequencing, and other technologies are not perfect, resulting in inconsistent expectations. Finally, there is, currently, a lack of clinical trials of circRNAs as biomarkers. Further studies on the biogenesis, degradation, and biological functions of circRNAs will deepen our understanding of AIDs and help provide new insights into the diagnosis and treatment of AIDs. In the future, circRNAs will hopefully become reasonable and efficient biomarkers of AIDs.

## Consent for Publication

All authors have agreed on the consent of the manuscript.

## Author Contributions

XZ performed the selection of literature, drafted the manuscript, and prepared the Figures. YZ, SX, and PC collected the related references and participated in discussions. JL designed this review and revised the manuscript. All authors contributed to this manuscript and read and approved the final manuscript.

## Conflict of Interest

The authors declare that the research was conducted in the absence of any commercial or financial relationships that could be construed as a potential conflict of interest.

## Abbreviations

AF: atrial fibrillation
AIDs: autoimmune diseases
AIRE: autoimmune regulator
ceRNA: competing endogenous RNA
circRNA: circular RNAs
EBV: Epstein–Barr virus
GWAS: genome-wide association study
IL-2 (6/8): interleukin-2(6/8)
MRE: miRNA response element
MS: multiple sclerosis
ncRNA: non-codingRNA
PBC: primary biliary cirrhosis
PBMCs: peripheral blood monouclear cells
PKR: double-stranded RNA (dsRNA)-dependent protein kinase
Pol II: RNA polymerase II
RA: rheumatoid arthritis
RBPs: RNA-binding proteins
RCMs: reverse complementary paired sequences
RF: rheumatoid factor
RHD: rheumatic heart disease
SA: splice acceptor sites
SD: splice donor sites
SLE: systemic lupus erythematosus
TNF-α: tumor necrosis factor-α.
